# Diagnosis and Management of Epileptic Encephalopathies in Children

**DOI:** 10.1155/2013/501981

**Published:** 2013-07-22

**Authors:** Puneet Jain, Suvasini Sharma, Manjari Tripathi

**Affiliations:** ^1^Division of Pediatric Neurology, Department of Pediatrics, All India Institute of Medical Sciences, New Delhi 110029, India; ^2^Department of Pediatrics, Lady Hardinge Medical College and Associated Kalawati Saran Children's Hospital, New Delhi 110001, India; ^3^Department of Neurology, Neurosciences Centre, All India Institute of Medical Sciences, New Delhi 110029, India

## Abstract

Epileptic encephalopathies refer to a group of disorders in which the unremitting epileptic activity contributes to severe cognitive and behavioral impairments above and beyond what might be expected from the underlying pathology alone, and these can worsen over time leading to progressive cerebral dysfunction. Several syndromes have been described based on their electroclinical features (age of onset, seizure type, and EEG pattern). This review briefly describes the clinical evaluation and management of commonly encountered epileptic encephalopathies in children.

## 1. Introduction

The term “epileptic encephalopathy” refers to a group of disorders in which the unremitting epileptic activity contributes to progressive cerebral dysfunction. This cannot be explained by the underlying etiology alone [1]. It may be progressive or have waxing-waning course. The underlying etiology is diverse. Their clinical and electroencephalographic (EEG) features mirror the specific age-related epileptogenic reaction of the immature brain. The various syndromes of epileptic encephalopathy are tabulated in Table 1. This review will briefly discuss the diagnosis and management of these syndromes according to the age of onset.

## 2. Early Infantile Epileptic Encephalopathies

This group of disorders comprises Ohtahara syndrome or early infantile epileptic encephalopathy (EIEE), early myoclonic encephalopathy (EME), and malignant migrating partial seizures in infancy. 

*Ohtahara syndrome* is a devastating epilepsy with onset ranging from intrauterine period to 3 months of age. The *tonic spasms* are the defining seizure type which are very frequent and occur in both sleep and wakeful states. Besides these, partial and rarely myoclonic seizures may be observed. The interictal EEG shows burst suppression pattern with no sleep-wake differentiation. The bursts last for 2–6 seconds alternating with periods of suppression lasting for 3–5 seconds. 

The underlying causes are heterogenous. The majority of cases are attributable to static structural brain lesions such as focal cortical dysplasia, hemimegalencephaly, and Aicardi syndrome [2, 3]. Few genetic mutations have been described but these are not specific for Ohtahara syndrome [4, 5]. These include mutations in the syntaxin binding protein-1 (*STXBP-1*) [6], Aristaless-related homeobox (*ARX*) [7], and* SLC25A22*-gene encoding a mitochondrial glutamate carrier [8]. An epileptic encephalopathy similar to Ohtahara syndrome, attributable to mutations in the *KCNQ2 *gene that encodes the voltage-gated potassium channel Kv7.2, has been recently described [9]. Though the condition appears similar to Ohtahara syndrome, subtle differences include progression with reduction in seizure frequency in *KCNQ2 *encephalopathy in comparison to Ohtahara syndrome which frequently evolves to West syndrome and the unusual transient basal ganglia imaging abnormalities in* KCNQ2* encephalopathy. 

The medical management of seizures is not rewarding. The dietary therapy has been tried with some success. Ishii et al. reported a favourable response of ketogenic diet in a male infant with Ohtahara syndrome who had failed multiple antiepileptic drugs [10]. Neurosurgery is sometimes favourable in selected cases of cerebral malformations [11]. The prognosis is uniformly poor with survivors left with severe psychomotor retardation. There may be age-dependent evolution to West syndrome and then subsequently to Lennox-Gastaut syndrome. 

*Early myoclonic encephalopathy* presents within the first 3 months of age and mostly within the neonatal period. The prenatal onset is known. Fragmentary, erratic myoclonia, partial seizures, and less frequently tonic spasms are seen. The interictal EEG shows burst suppression pattern more prominent during the sleep. The bursts last for 1–5 seconds with longer periods of suppression (3–10 seconds).  Table 2 shows differentiating features between EEG features of Ohtahara syndrome and early myoclonic encephalopathy.

Many of the reported cases are familial. The inborn errors of metabolism such as nonketotic hyperglycinemia, organic acidemia, Menkes disease, Zellweger syndrome, molybdenum cofactor deficiency [2], pyridoxine dependency [12], and genetic factors are the most important etiologies. The structural abnormalities are rarely found [2].

These seizures are often refractory to conventional antiepileptic drugs. A sequential therapeutic trial with pyridoxine, folinic acid, and pyridoxal phosphate should be instituted [13]. Limited success has been reported with ketogenic diet [14]. The prognosis is dismal. The EEG often evolves to atypical hypsarrhythmia which is transient or multifocal spike and sharp waves 3-4 months after the onset of the disease. 

The diagnosis of these epileptic encephalopathies begins with an EEG which should include both the sleep and wake states. A magnetic resonance imaging of the brain must be obtained to look for structural defects. A metabolic profile including blood ammonia, arterial blood gas, lactate, blood tandem mass spectrometry, and urine organic acid analysis must be obtained to look for inherited metabolic defects. Testing for* STXBP1* mutations may be considered in infants with Ohtahara syndrome, once brain malformations or inherited metabolic defects have been excluded [13].

*Epilepsy of infancy with migrating focal seizures,* is a rare, age-specific epileptic encephalopathy with a malignant course with onset in the first 6 months of age. It is characterized by a period of normal early development followed by nearly continuous migrating polymorphous focal seizure which are intractable, with subsequent psychomotor retardation and, in most children, progressive decline of head circumference percentile [15, 16].

Interictal EEGs show diffuse slowing of the background activity with multifocal epileptiform discharges. Ictal EEGs display paroxysmal discharges occurring in various regions in consecutive seizures in a given patient. They start in one region and progressively involve the adjacent areas. The area of ictal onset shifts from one region to another and from one hemisphere to the other, with occasional overlapping of consecutive seizures.

The etiology is largely unknown. SCN1A [17, 18] and phospholipase C*β*1 (PCB1) [19] gene mutations have been described in few patients. The seizures are markedly pharmacoresistant to conventional antiepileptic drugs. Potassium bromide [20] and stiripentol [21] have been tried with some success. The ketogenic diet has been unsuccessful [22].

## 3. West's Syndrome

West's syndrome was first described by West in 1841 [23] and is characterized by epileptic spasms or “*salaam attacks,*” hypsarrhythmia on EEG, and developmental delay or regression. The typical onset is between 3 and 12 months of age. The epileptic spasms are clusters of sudden, brief (0.2–2 seconds), diffuse or fragmented, and tonic contractions of axial and limb muscles. This may be accompanied by cry, laughter, or autonomic changes. It may be flexor (most common), extensor, mixed, or subtle. They usually occur in arousal and in alert states [24].

**Figure 1:**
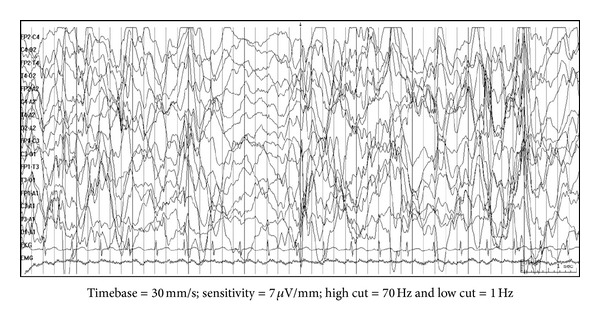
EEG findings in classical hypsarrhythmia: the background is chaotic with bursts of bilateral asynchronous high-amplitude slow waves interspersed with spikes followed by electrodecremental response.

**Figure 2:**
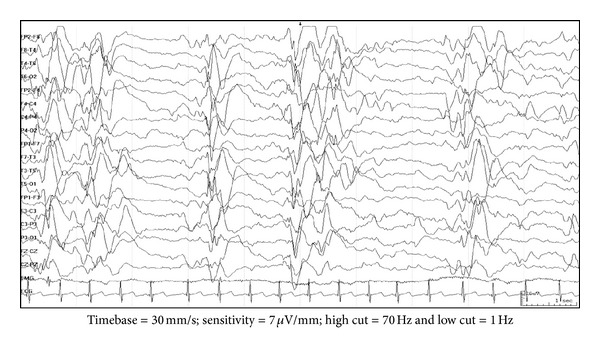
EEG findings in hypsarrhythmia (burst-suppression) variant: there are bursts of bilateral asynchronous high-amplitude slow waves interspersed with spikes followed by generalized voltage attenuation.

Hypsarrhythmia is the classical interictal EEG finding and is characterized by chaotic background with nearly continuous random asynchronous high-voltage slow waves and spikes arising from multiple foci (Figure 1) [25]. Many variations have been described [26]. These include increased interhemispheric synchronization, consistent voltage asymmetries, consistent focus of abnormal discharge, episodes of generalized/regional or lateralized voltage attenuation (Figure 2), primarily high-voltage bilaterally asynchronous slow wave activity with relatively little epileptiform abnormalities. Both classic and variant hypsarrhythmia have the same prognosis. The variation occurs because of sleep state, etiology, disease course, and treatment. 

The EEG becomes fragmented and more synchronized during nonrapid eye movement (NREM) sleep and relatively normalizes during rapid eye movement (REM) sleep. Ictal EEG patterns are variable and may comprise classical electrodecremental pattern, high-voltage generalized slow-wave, or low-amplitude fast activity [24].

The etiology is diverse. West's syndrome has been classically classified into symptomatic (identifiable neurological insult), cryptogenic (probably symptomatic but with no known etiology), and idiopathic (normal premorbid development and unknown etiology) forms. The classification as per new ILAE classification [1] is shown in Table 3. Recently, a genetic and biologic classification has been suggested [27]. Thus, a thorough clinical evaluation followed by appropriate neuroimaging and genetic and metabolic work-up is warranted in a child with West's syndrome.

Adrenocorticotrophin hormone (ACTH) is the drug of choice for short-term treatment of epileptic spasms. Low-dose ACTH may be equally effective as high dose ACTH [28]. Oral steroids may also be an alternative, especially in resource-constrained settings [29]. Vigabatrin is a second-line drug except in children with tuberous sclerosis complex where it is the preferred drug over ACTH. Pyridoxine and biotin trial should always be considered in refractory spasms or when clinically indicated. The ketogenic diet has also shown to be beneficial [30–32]. Resective neurosurgery may be warranted in refractory cases with unilateral or focal congenital or early acquired cortical lesions [33]. Total callosotomy may be considered in children with persistent drop attacks [34].

The prognosis is guarded and is governed by the underlying etiology and the treatment. The affected children are left with variable psychomotor retardation, epilepsy, or psychiatric disorders [24].

## 4. Late Infantile Epileptic Encephalopathy

This entity has been proposed by Nordli et al. [35, 39]. The onset is beyond one year of age with classical *myoclonic-tonic* seizures. The tonic component is longer than the infantile spasms and shorter than that seen in Lennox-Gastaut syndrome. There may be associated myoclonic seizures, epileptic spasms, and atonic seizures. The interictal EEG shows disorganized high-amplitude slow background with multifocal spikes more pronounced during the sleep. The response to the conventional antiepileptic drugs is poor with some response to hormonal therapy and ketogenic diet. The prognosis is guarded.

## 5. Dravet Syndrome

It was first described by Dravet in 1978 as severe myoclonic epilepsy of infancy (SMEI) [40]. The onset is usually between 5 and 8 months of age with frequent, prolonged febrile unilateral clonic convulsions with alternating pattern in a previously normal child. Nonfebrile seizures may also be present. This stage is followed by emergence of multiple seizure types (myoclonic, atypical absences and complex focal seizures) which frequently progress to status epilepticus and associated severe psychomotor deterioration. The relentless progression stops at around 10–12 years of age with decrease in seizure frequency and persisting neurologic sequalae [41].

The interictal EEG is normal initially. In some cases, generalized photoparoxysmal responses and rhythmic theta (4-5 Hz) activity may be seen in centroparietal areas and vertex. Soon, the EEG deteriorates with background slowing, asymmetric paroxysms of generalized polyspike/spike-slow-wave discharges and multifocal epileptiform abnormalities. Photic, pattern, and eye closure sensitivity may be present [24, 42].

The children with borderline SMEI or intractable childhood epilepsy with generalized tonic clonic seizures (ICEGTCS) may lack myoclonic seizures or generalized spike-and-wave activity [43].

Mutations in the SCN1A gene encoding the alpha-1 subunit of the sodium channel are detectable in 70–80% of patients with Dravet syndrome [44]. Other reported mutations include mutations in genes GABARG2 (encoding *γ*2 subunit of GABA_A_ receptor), SCN1B and protocadherin 19 (PCDH19) genes [44, 45]. 

Seizures are usually refractory. Drugs like carbamazepine, phenytoin, and lamotrigine are contraindicated. Stiripentol in conjunction with clobazam or valproate has recently been licensed for use in Dravet syndrome [46]. Early initiation of ketogenic diet has been advocated [47]. Avoidance of hyperthermia and stress is critical. 

## 6. Lennox-Gastaut Syndrome

Lennox-Gastaut syndrome (LGS) is a severe form of epileptic encephalopathy with onset between 1 and 8 years of age, mainly between 2 and 5 years of age. It is characterized by intractable polymorphic seizures including tonic, atypical absence, atonic and myoclonic seizures. “Drop attacks,” tonic or atonic, seen in 50% children, are a nightmare for the family and frequently causes injuries [48]. Two-thirds of the patients may have nonconvulsive status epilepticus [49]. Twenty percent of children have history of epileptic spasms [50]. The cognitive deterioration/stagnation is common and fluctuates with the seizure frequency. 

**Figure 3:**
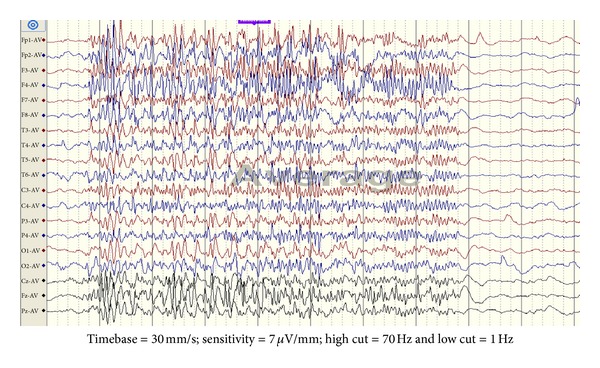
Generalized paroxysmal fast activity: there are bursts of bilateral synchronous high-frequency low-amplitude activity lasting for 7 seconds with sudden onset and resolution.

The pathognomonic interictal EEG finding is bilateral, synchronous, and slow spike-and-wave discharges (1.5–2.5 Hz) with frontocentral voltage dominance with abnormal background. Paroxysmal fast activity (Figure 3) of bilateral synchronized bursts of 10–20 Hz frontally dominant activity lasting for few seconds is also seen. It may be an ictal correlate of a tonic seizure, especially if prolonged. Focal discharges are common. NREM sleep dramatically enhances all the paroxysmal abnormalities. Other abnormalities include sleep fragmentation of the slow spike-and-wave bursts, polyspike discharges, pseudoperiodic appearance, diffuse voltage attenuation, focal and multifocal spikes and sharp waves, diffuse background slowing, abnormal sleep architecture with reduced or absent REM sleep, and severe background disorganization with a quasihypsarrhythmic pattern in some patients [51].

The etiology of Lennox-Gastaut syndrome is heterogenous and similar to epileptic spasms (see Table 2). One-third of children have no antecedent history or evidence of cerebral pathology [24].

Lowering the frequency of serious/disabling seizures like drop attacks, minimizing daytime seizures, and minimizing adverse effects of antiepileptic drugs may be a realistic management goal in children with Lennox-Gastaut syndrome. Valproate and clobazam are the preferred drugs. Levetiracetam, rufinamide, lamotrigine, topiramate, and zonisamide are the second-line drugs. Steroids and intravenous immunoglobulins may be indicated during periods of increased seizure frequency or status epilepticus [24, 36, 52–54]. The ketogenic diet is a useful alternative and may be used early in the management [55]. Nonpharmacological therapies also include vagus nerve stimulation [56, 57], electrical stimulation of centromedian thalamic nuclei [58], and complete or partial callosotomy. 

The prognosis is guarded with more than 80% children having persistent epilepsy and severe neurocognitive sequalae. Normal development prior to onset of seizures, normal neuroimaging, near normal background on EEG, faster generalized spike-wave-activity, and activation of generalized spike-wave-activity by hyperventilation may predict favourable outcome [24].

### 6.1. Case Study 1

A 7-year-old boy, a known case of Lennox-Gastaut syndrome secondary to perinatal asphyxia, presented with flurry of seizures (tonic and atypical absences). He was on 40 mg/kg/day valproate, 3 mg/kg/day lamotrigine, and 40 mg/kg/day levetiracetam. He had partial response to modified atkins diet in the past but had discontinued the diet due to poor compliance. In the emergency room, he was administered intravenous diazepam. After 10 minutes of diazepam administration, there was marked increase in the frequency of prolonged tonic spasms with cardiorespiratory compromise. The EEG showed frequent bursts of prolonged generalized paroxysmal fast activity with diffuse delta waves in between.

*Learning Point*. Intravenous benzodiazepines may result in paradoxical precipitation of tonic status in patients with LGS and hence should be used with caution in such patients.

## 7. Epileptic Encephalopathy with CSWS Including LKS

### 7.1. Landau-Kleffner Syndrome

This syndrome was first described by Landau and Kleffner [59]. The peak onset is between 5 and 7 years of age with verbal auditory agnosia in a previously normal child. The language function continues to deteriorate and the course can be gradually progressive or fluctuating. All types of aphasia can occur. Some children may become mute. Mild behavioral abnormalities are common. Seizures occur in 75% children. They are infrequent and usually nocturnal. Semiologies may include generalized tonic-clonic, focal motor, atypical absences, head drops, and subtle seizures. 

The EEG is characterized by mainly posterior temporal (vertical dipole) epileptiform discharges. These discharges can be multifocal, unilateral, or bilateral and markedly activated by NREM sleep [60]. They may continue into the REM sleep, a differentiating feature from epilepsy with CSWS [24].

The main aim of the treatment is to reduce or eliminate the epileptiform discharges. Valproate, benzodiazepines, levetiracetam, ethosuximide, and sulthiame are the most effective drugs [61]. Poor responders may be treated with ACTH or prednisolone. Prolonged oral steroids may be required as relapses are common on withdrawal. Steroids may be administered early in the course of the illness. The role of intravenous immunoglobulins is unclear [62]. Favourable results have been reported with ketogenic diet in small studies [63]. For medically refractory cases, multiple subpial transection including the Wernicke area has been used with some success especially if electrophysiologic lateralization can be demonstrated [64, 65].

The seizures and epileptiform abnormalities remit by the age of 15 years. The majority of children are left with permanent language dysfunction. The earlier the onset of LKS, the worse the prognosis with regard to the language function. 

### 7.2. Epileptic Encephalopathy with Continuous Spike-and-Wave during Sleep

The onset of this epileptic encephalopathy is between 2 months and 12 years of age with a peak at 4–7 years of age. The preceding neurodevelopment is normal in 50% children. Seizures are the presenting symptom in 80% children and neuropsychological deterioration in the rest. The children present with infrequent, nocturnal seizures (simple or complex focal, generalized tonic-clonic or myoclonic seizures). The interictal EEG during wakefulness shows focal or multifocal epileptiform discharges with accentuation during NREM sleep [66]. The localization of discharges can be frontocentral, frontotemporal, centrotemporal, or frontal [67]. 

**Figure 4:**
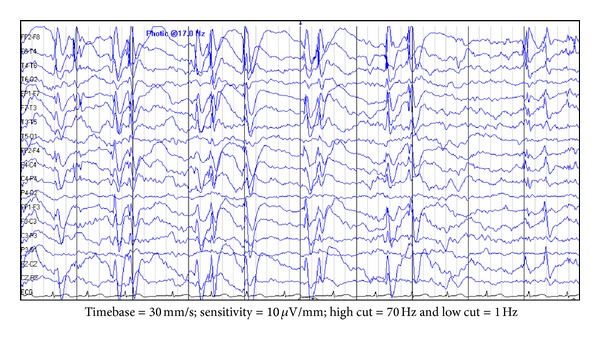
EEG findings in epileptic encephalopathy with continuous spike-and-wave during sleep: there is nearly continuous 1-2 Hz bilateral synchronized spike wave discharges during the sleep record.

After 1-2 years, there is increase in seizure frequency with emergence of new seizure types (absence or atonic seizures, negative myoclonus). This is associated with the appearance or deterioration of neurocognitive status. The symptoms depend on the predominant site of epileptiform discharges. Mainly, frontal CSWS affects the cognitive and executive functioning, and temporal-predominant-CSWS affects the linguistic function [24]. The interictal EEG during wakefulness shows more pronounced abnormalities. During NREM sleep, EEG shows continuous/nearly continuous, bilateral, 1.5–3 Hz, frontally predominant, and spike-wave-discharges (CSWS) which may be asymmetric or focal (Figure 4). They are also known as electrical status epilepticus during sleep (ESES) [68]. The spike wave index (SWI), a measure of the frequency of spiking in the EEG tracing, is usually more than 85%. EEG during REM sleep shows disappearance of ESES pattern. 

This stage is followed by clinicoelectroencephalographic remission, usually 2–7 years after the onset. The majority of the children, however, are left with residual moderate-to-severe neurocognitive deficits. 

The etiology is unknown. Abnormal neuroimaging is seen in 30–59% cases [66, 69, 70] and may include cerebral atrophy, perinatal vascular insults, and cerebral malformations. The evolution from benign childhood focal epilepsies to ESES is also reported [71].

Early initiation of steroids/ACTH is usually recommended. Intravenous immunoglobulins also have shown promising results [62]. The antiepileptic drugs, used for LKS, are usually effective. Limited response has been demonstrated with ketogenic diet [72]. Epilepsy surgery may be considered in medically refractory cases with focal lesions on neuroimaging or focal EEG findings. Hemispherectomy and focal resective epilepsy surgery may be beneficial for children with ESES with structural etiology [73]. With the encouraging results in the children with LKS, multiple subpial transections may be beneficial for the cognitive impairment and behavioural problems seen in epileptic encephalopathy with continuous spike-and-wave during sleep [74]. 

## 8. Atypical Benign Partial Epilepsy of Childhood 

This syndrome is also known as *LGS transient* or *pseudo-Lennox syndrome*. The onset is at 2–6 years of age in previously normal child with clusters of atonic and nocturnal focal “Rolandic-like” seizures. Variable cognitive involvement may be seen during periods of active seizures. The interictal EEG shows centrotemporal spikes (horizontal dipole) and generalized spike-and-wave discharges. The centrotemporal spikes may be seen in trains and may be associated with frontocentral and centroparietal spikes [75]. The interictal magnetoencephalography has localized the clusters of spike sources around the Rolandic-sylvian fissures [76]. This is in contrast to the findings in Rolandic epilepsy where the clusters of spike sources have been localized along the Rolandic region with orientation vertical to the central sulcus [77].

Similar condition may be induced by lamotrigine or carbamazepine in few children with rolandic and Panayiotopoulos syndromes [78, 79]. Some authors consider it as a mild form of epilepsy with continuous spike-and-wave during sleep (CSWS) [36]. It is still debatable whether this entity is a separate clinical entity or part of a continuum related to rolandic epilepsy [80].

The features like an earlier age of onset, frequent atonic seizures, more frequent and prolonged focal seizures, and prominent associated behavioural problems may differentiate this entity from rolandic epilepsy [75].

Seizures are usually refractory to conventional treatment but usually remit by adolescence. The long-term neurocognitive outcome is usually favourable [24, 48]. 

### 8.1. Case Study 2

A 5-year-old, developmentally normal boy presented with multiple episodes of atypical absences, atonic seizures, and nocturnal focal seizures for the last 3 months. He had mild behavioural complaints. The examination was unremarkable. A diagnosis of possible LGS was made, and the boy was initiated on valproate and clonazepam. An interictal EEG revealed normal background with frequent centro-temporal spikes. The diagnosis was revised to atypical benign partial epilepsy in view of the clinic EEG features.

*Learning Point*. LGS may be a clinical differential of atypical benign partial epilepsy. However, the lack of tonic seizures or developmental delay and normal awake EEG background activity differentiates atypical benign partial epilepsy from LGS.

## 9. Conclusions

Epileptic encephalopathies start at an early age and manifest with seizures, which are usually intractable, aggressive EEG paroxysmal abnormalities and severe neurocognitive deficits. The clinicoelectroencephalographic features are age related and depend on the structural and functional maturity of the brain. Their recognition and appropriate management are critical.

## Figures and Tables

**Table 1:** Epileptic encephalopathies.

Recognized syndromes	
Ohtahara syndrome	
Early myoclonic encephalopathy	
West syndrome	
Dravet syndrome	
Lennox-Gastaut syndrome	
Epileptic encephalopathy with continuous spike-and-wave during sleep (CSWS)	
Landau-Kleffner syndrome (LKS)	
Proposed	
Epilepsy of infancy with migrating focal seizures [15, 16]	
Late infantile epileptic encephalopathy [35]	
Atypical benign partial epilepsy of childhood [36]	
Hypothalamic epilepsy [37]	
Myoclonic encephalopathy in nonprogressive disorders [38]	

**Table 2:** EEG features of Ohtahara syndrome and early myoclonic encephalopathy.

Feature of burst-suppression pattern	Ohtahara syndrome	Early myoclonic encephalopathy
Appearance	Usually seen at the onset of the disease	Seen later; most distinct at 1–5 months of age
Disappearance	Within the first 6 months	Persists for longer periods
State in which it presents	Both sleeping and waking states	Exclusively present or enhanced during sleep
Burst-to-burst intervals	Shorter	Longer
Evolution to hypsarrhythmia	Frequent	May be a transient feature

**Table 3:** Classification of West syndrome.

Structural/metabolic	
Pre-, peri-, and postnatal cerebral ischemia	
Cerebral malformations	
Neuro-infections sequalae	
Neurocutaneous syndromes: tuberous sclerosis, incontinentia pigmenti	
Hypothalamic hamartoma	
Inborn errors of metabolism: biotinidase deficiency and other organic aciduria, phenylketonuria, mitochondrial disorders, Menkes disease, nonketotic hyperglycinemia, and antiquitin deficiency	
Genetic	
Genetic: CDKL-5, MeCP 2, ARX, STXBP-1, SPTAN1, and PLC-*β*1	
Chromosomal disorders: down syndrome, 1p36 deletion, and Pallister-Killian syndrome	
Unknown	
